# Supplementary material for: HSF1 Stimulates Glutamine Transport by Super-Enhancer-Driven lncRNA LINC00857 in Colorectal Cancer
Source: Cancers (Basel). 2022 Aug 9;14(16):3855. doi: 10.3390/cancers14163855 (PMC9406190; doi:10.3390/cancers14163855)
Supplement: Supplementary file 1 [file cancers-14-03855-s001.zip › cancers-1821523-supplementary.pdf]

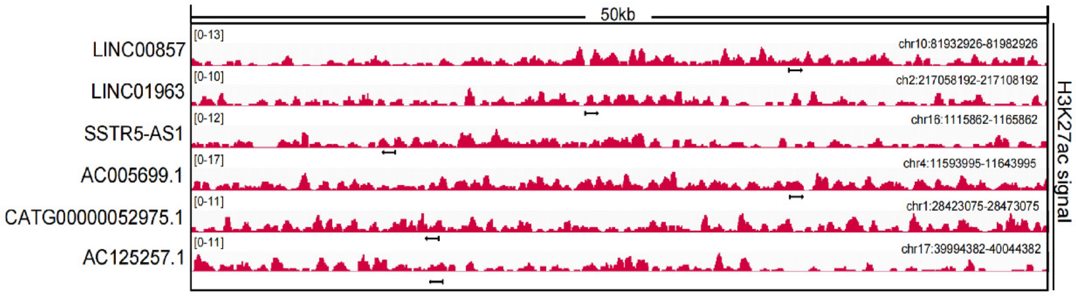

Figure S1. Gene tracks of H3K27ac ChIP-seq occupancy at representative HSF1-associated lncRNA gene loci in HCT116 cell.

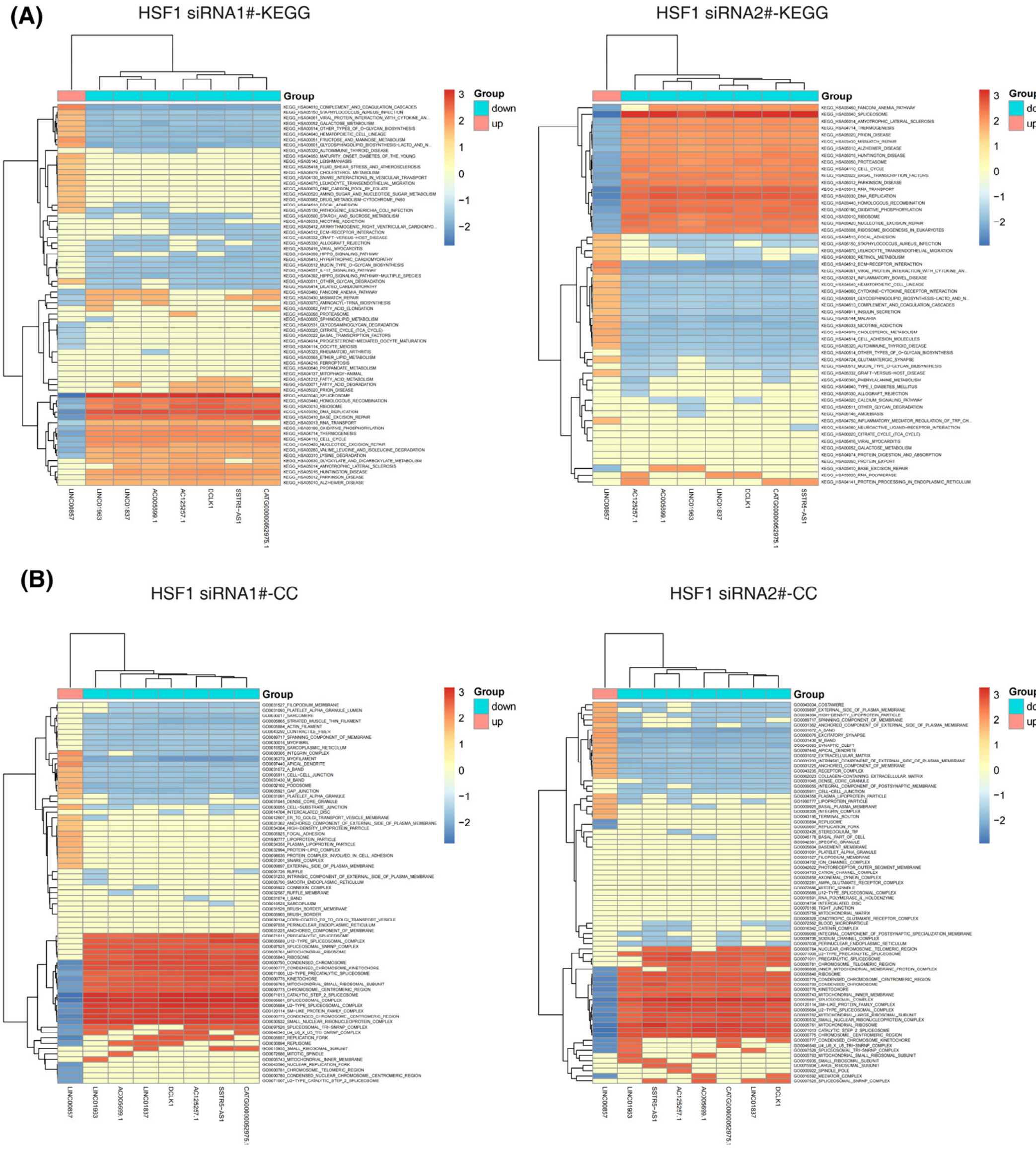

Figure S2. Gene Set Enrichment Analysis (GSEA) on 8 HSF1-associated downregulated lncRNAs. (KEGG: kyoto encyclopedia of genes and genomes, CC: Cellular component).

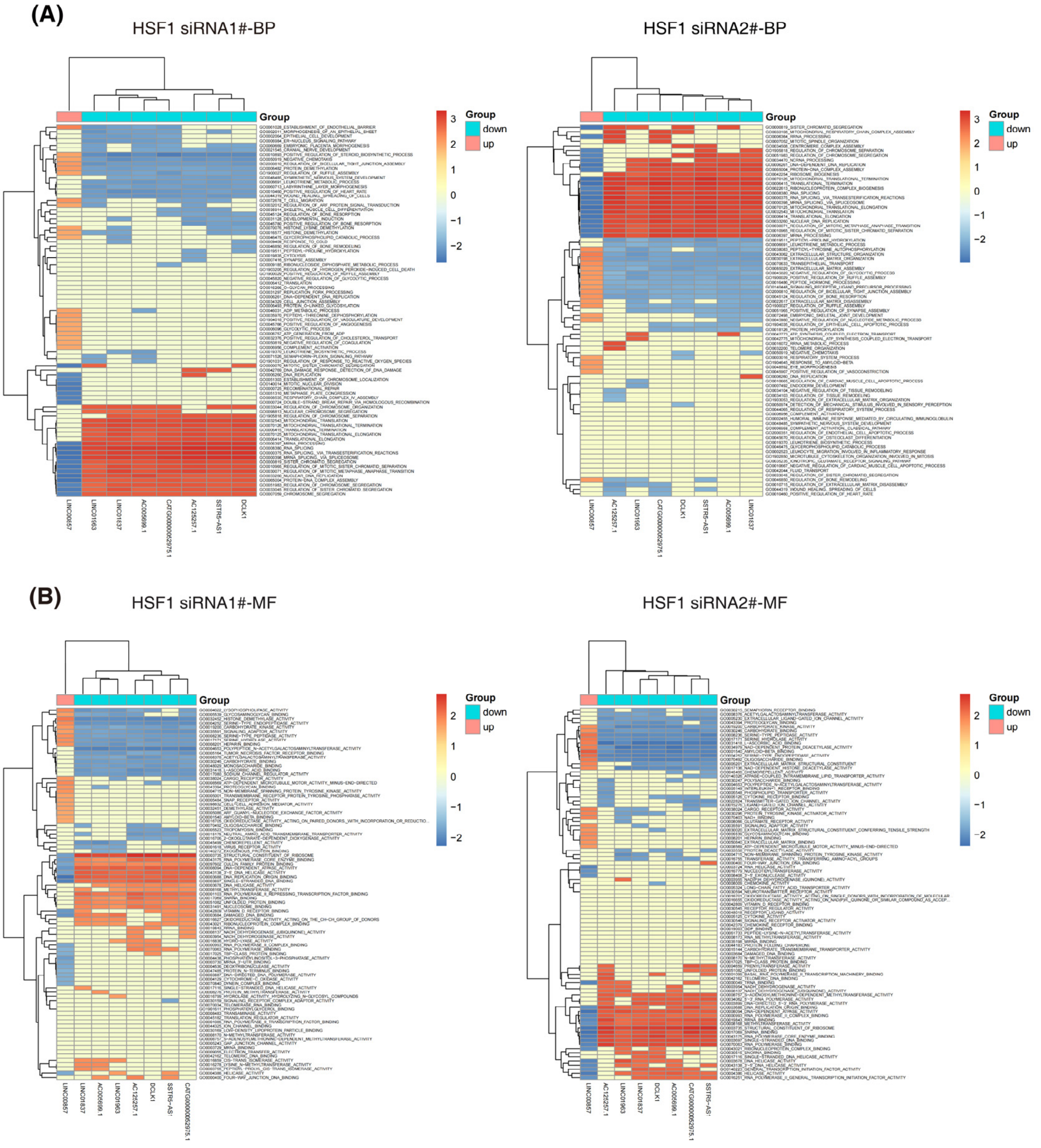

Figure S3. Gene Set Enrichment Analysis (GSEA) on 8 HSF1-associated downregulated lncRNAs. (BP, Biological process, MF, Molecular function).

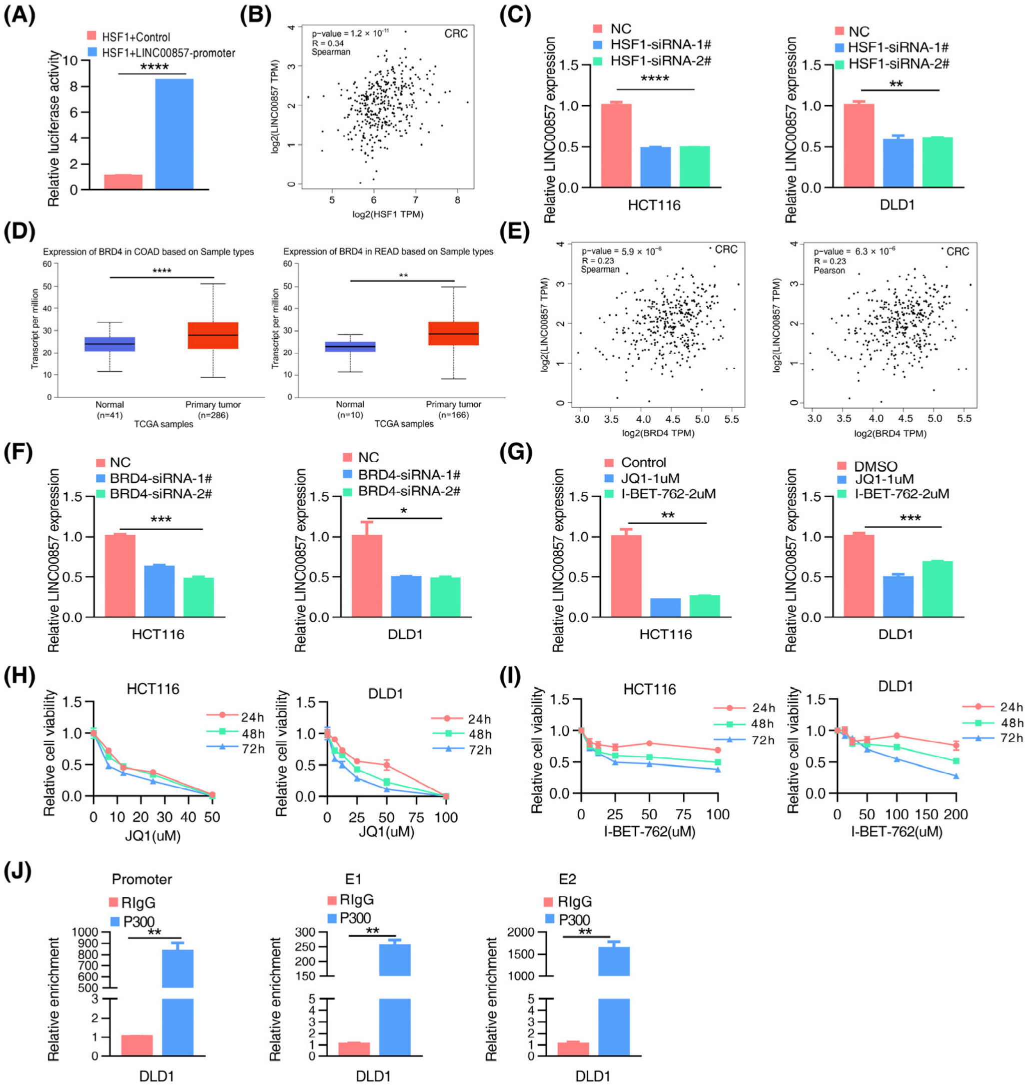

Figure S4. The effect of HSF1 and super enhancer on LINC00857 expression. (A) The relative luciferase activity after transfection HSF1 and LINC00857 promoter. (B) Correlation between LINC00857 and HSF1 expression using GEPIA database. (C) Relative LINC00857 expression after HSF1 knockdown. (D) Comparison of BRD4 expression levels between normal and tumor in CRC by UALCAN database (COAD, colon cancer, READ, rectum cancer). (E) Correlation between LINC00857 and BRD4 expression using GEPIA database. (F) Relative LINC00857 expression after BRD4 knockdown by qPCR assays. (G) Relative LINC00857 expression after the treatment with JQ1 (1μM) or iBET-762 (2μM) by qPCR assays. (H-I) HCT116 and DLD1 cells were treated with JQ1 and I-BET-762, and the relative cell viability was determined by CCK8 assays. (J) The relative enrichment level of P300 to LINC00857 promoter and enhancers by ChIP-qPCR assays. All data represents mean ± SD, n=3. * $p < 0.05$; ** $p < 0.01$; *** $p < 0.001$; **** $p < 0.0001$.

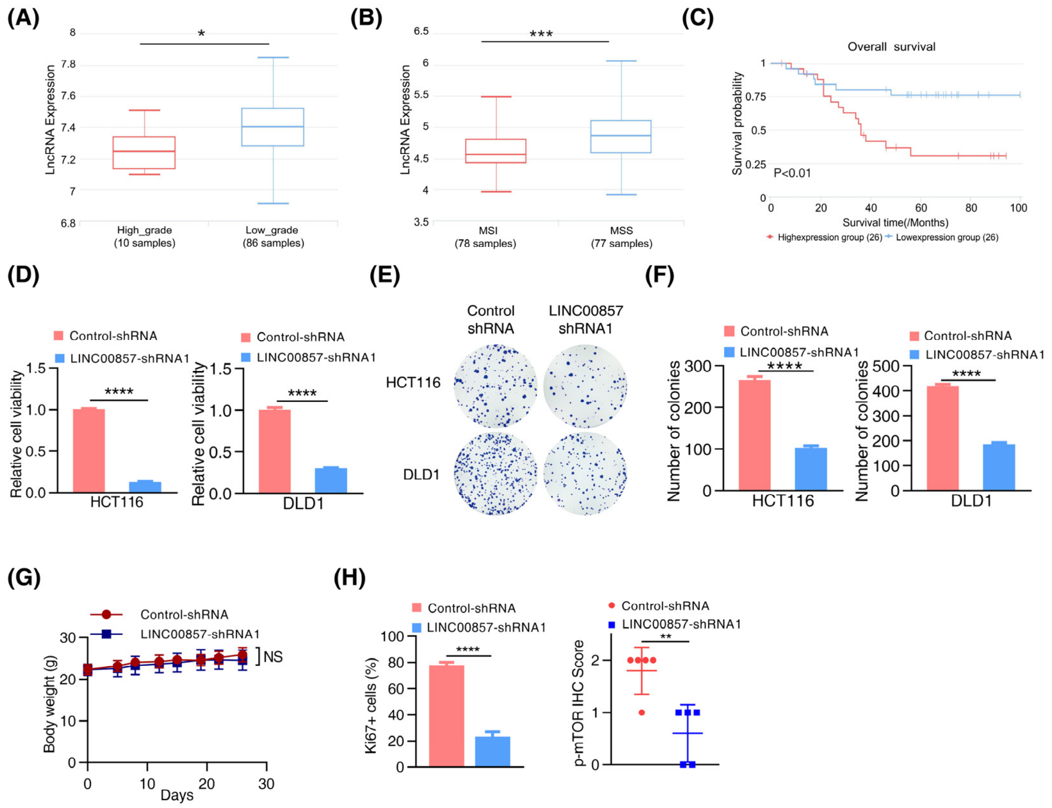

Figure S5. LINC00857 is upregulated and beneficial for CRC carcinogenesis. (A-B) Analysis of LINC00857 expression levels by data from lnCAR database (A. CR_S101, B. CR_S104). (C) The prognostic value of LINC00857 in COAD (colon cancer) using the lnCAR database (CR_01). (D-F) Relative cell viability and colony formation assays was measured after LINC00857 knockdown by shRNA. (G) The body weight in different groups in nude mice assays. (H) Quantification for IHC analysis of Ki67 and p-mTOR activity in nude mice assays. All data represents mean ± SD, n≥3. * $p < 0.05$; ** $p < 0.01$; *** $p < 0.001$; **** $p < 0.0001$.

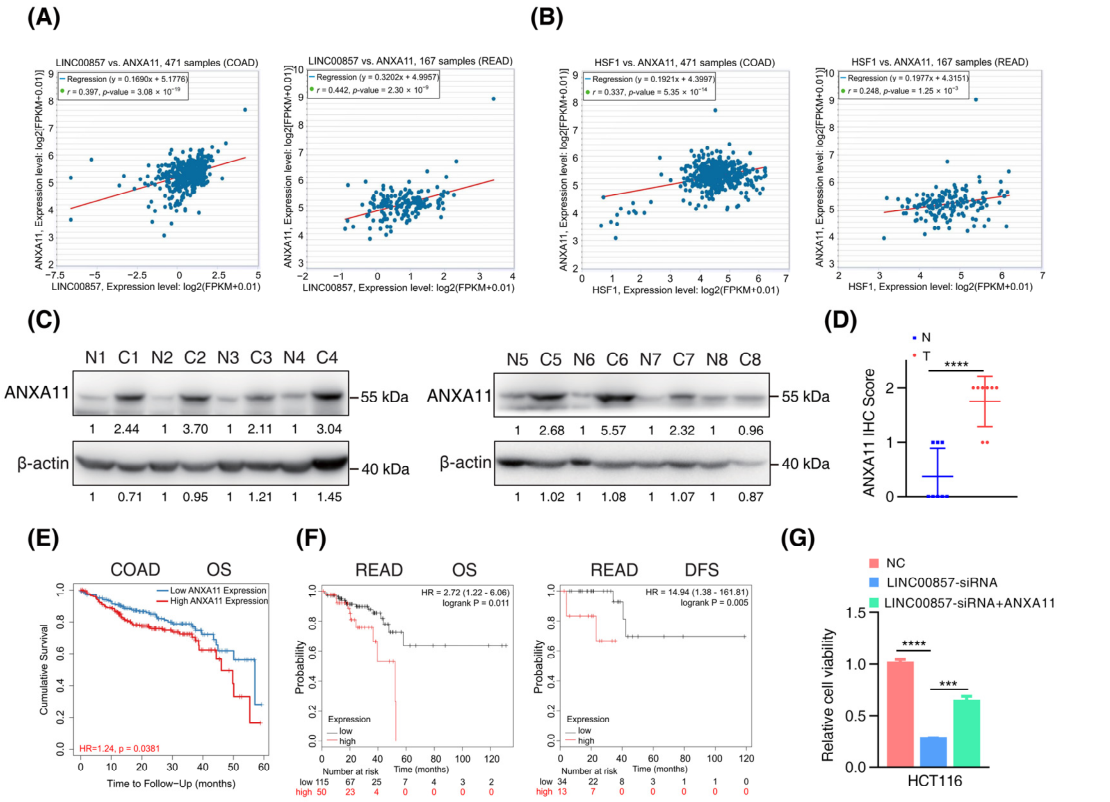

Figure S6. The expression and prognostic value of ANXA11 in CRC. (A-B) Correlation between LINC00857, ANXA11 and HSF1 expression using Starbase database. (C) The increased expression of ANXA11 in colorectal tumor tissues (n=8) compared to matched normal tissues. (D) Quantification for IHC analysis of ANXA11 in paired colorectal tumor tissues. (E) The association between ANXA11 expression and overall survival (OS) in COAD (colon cancer) was analyzed by TIMER database. (F) Kaplan-Meier survival curves for OS and DFS of patients with high and low ANXA11 expression in READ (rectum cancer) (OS, overall survival; DFS, disease free survival). (G) The effect of LINC00857 knockdown on cell viability in HCT116 cells treated with exogenous ANXA11 was analyzed by CCK-8 assays. All data represents mean ± SD, n≥3. * $p < 0.05$; ** $p < 0.01$; *** $p < 0.001$; **** $p < 0.0001$.

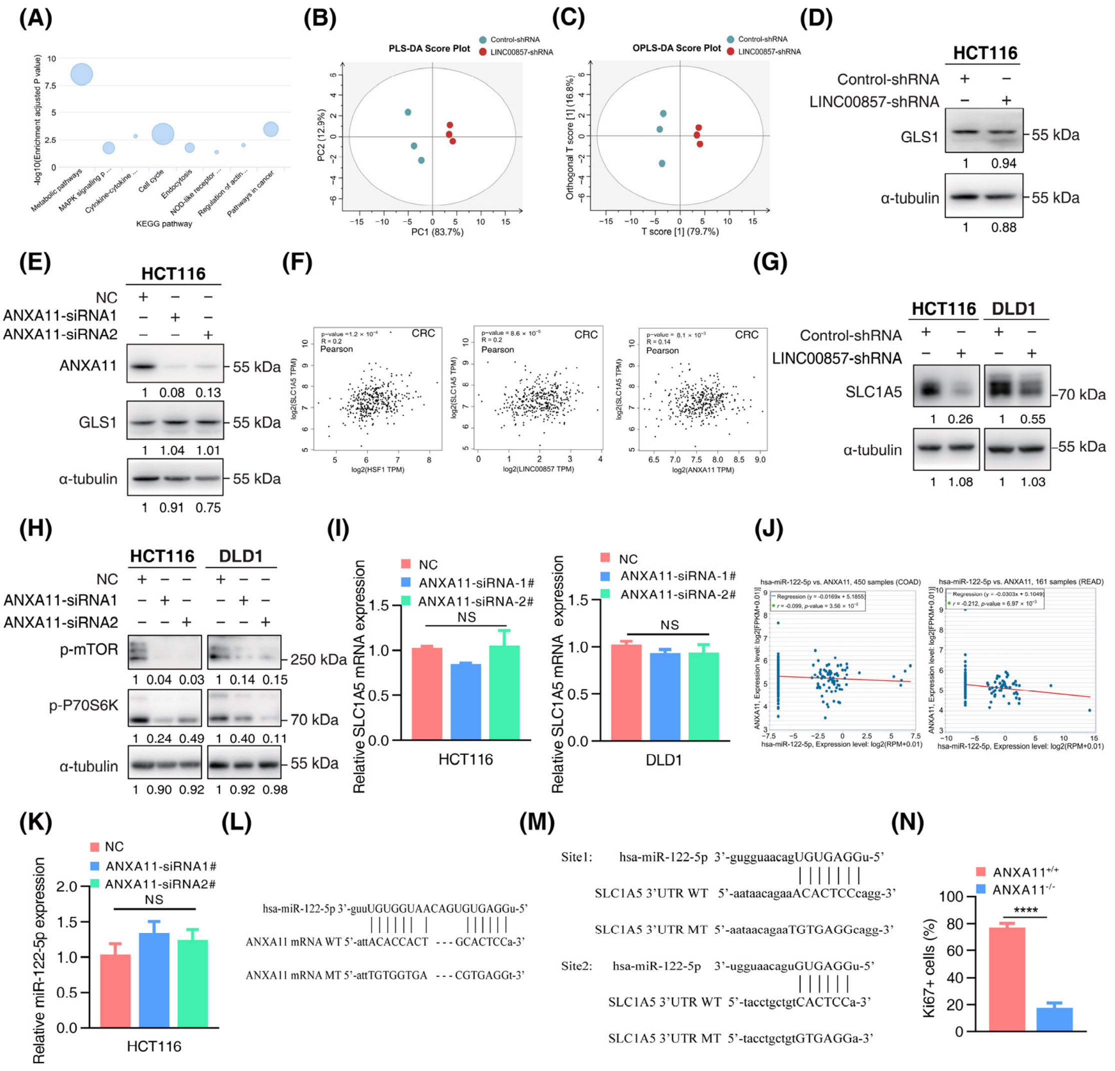

Figure S7. The effect of LINC00857-ANXA11 axis on glutamine metabolism. (A) KEGG (kyoto encyclopedia of genes and genomes) pathway analysis of LINC00857 by lnCAR database. (B-C) Score plot of partial least squares discriminant analysis (PLS-DA) and orthogonal partial least squares discriminant analysis (OPLS-DA) between control and LINC00857 knockdown group after LC-MS (liquid chromatography mass spectrometry) analysis. (D-E) The effect of LINC00857 and ANXA11 knockdown on GLS1 expression was explored by western blotting. (F) Correlation between HSF1-LINC00857-ANXA11 axis and SLC1A5 expression using GEPIA database. (G) The effect of LINC00857 knockdown on SLC1A5 expression was explored by western blotting. (H) The effect of ANXA11 knockdown on p-mTOR activity was explored by western blotting. (I) The effect of ANXA11 knockdown on SLC1A5 mRNA expression was analyzed by qPCR. (J) The scatter plot of the relationship between hsa-miR-122-5p and ANXA11 expression according to StarBase data. (K) The effect of ANXA11 knockdown on miR-122-5p expression was analyzed by qPCR. (L-M)

Schematic representation of the predicted target site for miR-122-5p in ANXA11 mRNA and SLC1A5 3'UTR. (N) Quantification for IHC analysis of Ki67 in $ANXA11^{+/+}$ and $ANXA11^{-/-}$ mice colorectal tissues. All data represents mean ± SD, n≥3. * $p < 0.05$; ** $p < 0.01$; *** $p < 0.001$; **** $p < 0.0001$.

Table S1. siRNA sequences.

Name	siRNA sequences
Negative control	S: UUCUCCGAACGUGUCACGUTT
	AS: ACGUGACACGUUCGGAGAATT
HSF1-siRNA 1#	S: CCUGAAGAGUGAAGACAUATT
	AS: UAUGUCUUCACUCUUCAGGTT
HSF1-siRNA 2#	S: GGACAAGAAUGAGCUCAGUTT
	AS: ACUGAGCUCAUUCUUGUCCTT
ANXA11-siRNA 1#	S: AGGCCAUCAUUGACUGCCUTT
	AS: AGGCAGUCAAUGAUGGCCUTT
ANXA11-siRNA 2#	S: GGUGAAAUGUCUCAAGAAUTT
	AS: AUUCUUGAGACAUUUCACCTT
BRD4-siRNA 1#	S: CCGUGAUGCUCAGGAGUUUTT
	AS: AAACUCCUGAGCAUCACGGTT
BRD4-siRNA 2#	S: AGCUGAACCUCCCUGAUUATT
	AS: UAAUCAGGGAGGUUCAGCUTT
LINC00857-siRNA 1#	S: GGCUAUGUGCUGUGAACAATT
	AS: UUGUUCACAGCACAUAGCCTT
LINC00857-siRNA 2#	S: GGUAUUAGUGGGUGAAUAUTT
	AS: AUAUUCACCCACUAAUACCTT
has-miR-122-5p mimics	S: UGGAGUGUGACAAUGGUGUUUG
	AS: AACACCAUUGUCACACUCCAUU
miRNA inhibitor NC	CAGUACUUUUGUGUAGUACAA
has-miR-122-5p inhibitors	CAAACACCAUUGUCACACUCCA
sh-NC	TTCTCCGAACGTGTCACGT
sh-LINC00857	GGCTATGTGCTGTGAACAA

Table S2. Primer sequences.

Name	sequences
HSF1-F	ACGGAGTTCCAGCACCCA
HSF1-R	CGCCACAGAGCCTCATTCT
a-Tubulin-F	GAAGCAGCAACCATGCGTGA
a-Tubulin-R	AAGGAATCATCTCCTCCCCCA
LNC00857-promoter-F	GTAGAGACGGGGTTTCACTATG
LNC00857-promoter-R	GCCTGTAATCCCAGCACTTT
LNC00857-E1-F	AATACTGGAGCTGGAAAGGGC
LNC00857-E1-R	TGCTCTCTGGGTCTCAGCTC
LNC00857-E2-F	GTAGCAGCTCCCAGGAATGTG
LNC00857-E2-R	GCGAGGCTTGGGGAGATTAAG
ANXA11-promoter-F	CGTGCCCAGTCCTCATCTTG
ANXA11-promoter-R	CAGGTGCCAGAGGCAGTG
LINC00857-F	AGAACGCGGTGTGAAGGAAA
LINC00857-R	TGAGCCCTGGGAAACAATGA
ANXA11-F	GTCCCCGTGGCCAGATCTAA
ANXA11-R	GGGTACAGGTTGGGCATGTT
BRD4-F	GTGGTGCACATCATCCAGTC
BRD4-R	TTGACTTCGGAGCCATCTCT
SLC1A5-F	TTTTTCCTGGTCACCACGCT
SLC1A5-R	TGGTAGAGTATGAGCGAAAGGC
has-miR-122-5p	TGGAGTGTGACAATGGTGTTTG
Actin-F	CACCAACTGGGACGACAT
Actin-R	ACAGCCTGGATAGCAACG

Table S3. ENCODE datasets.

Experiment	BigWig file	Target
ENCSR366OZN	ENCFF769FUM	H3K27ac
ENCSR955TUF	ENCFF665WBU	H3K27ac
ENCSR190DLX	ENCFF515EWP	H3K27ac
ENCSR856IMP	ENCFF660UEY	H3K27ac
ENCSR984BDR	ENCFF849LFK	H3K27ac
ENCSR853CTO	ENCFF142EKL	H3K27ac

Table S4. The clinicopathological parameters of CRC patients (n=8)

Patients	Ages	Gender	Location	Stage (T)	Stage (N)	Differentiation
Patient 1	53	Female	Rectum	T3	N1	High
Patient 2	51	Female	Rectum	T2	N0	High
Patient 3	67	Male	Rectum	T3	N1	High
Patient 4	58	Male	Colon (left)	T3	N1	Moderate
Patient 5	62	Male	Colon (left)	T3	N1	Moderate
Patient 6	61	Male	Colon (right)	T3	N2	Poor
Patient 7	52	Female	Colon (left)	T3	N2	Moderate
Patient 8	44	Female	Colon (left)	T3	N0	High

File S1: Original Western Blot figures

S-Figure 1

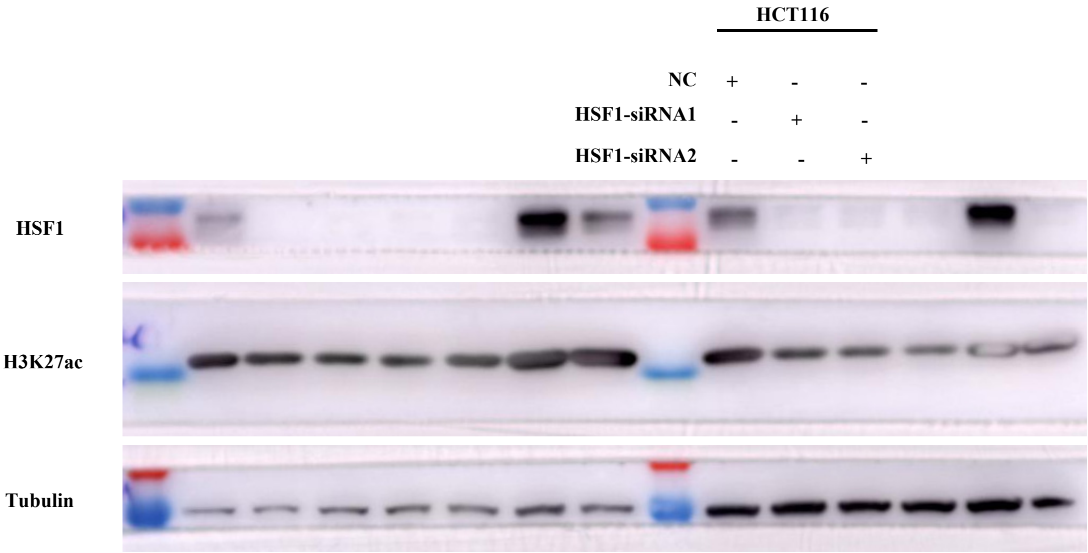

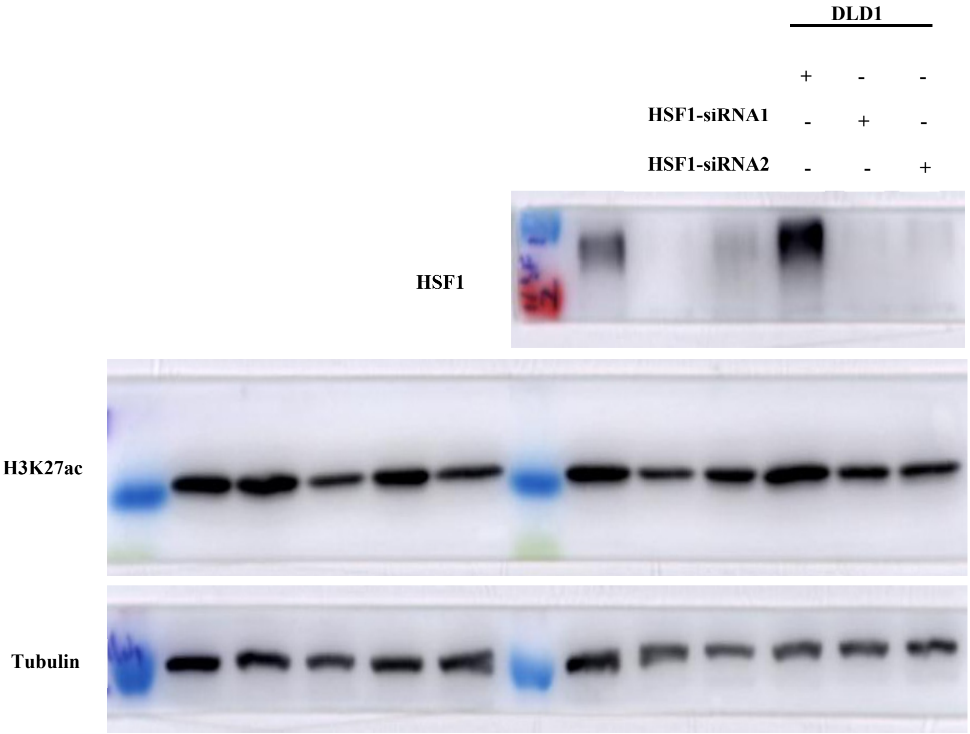

S-Figure 2

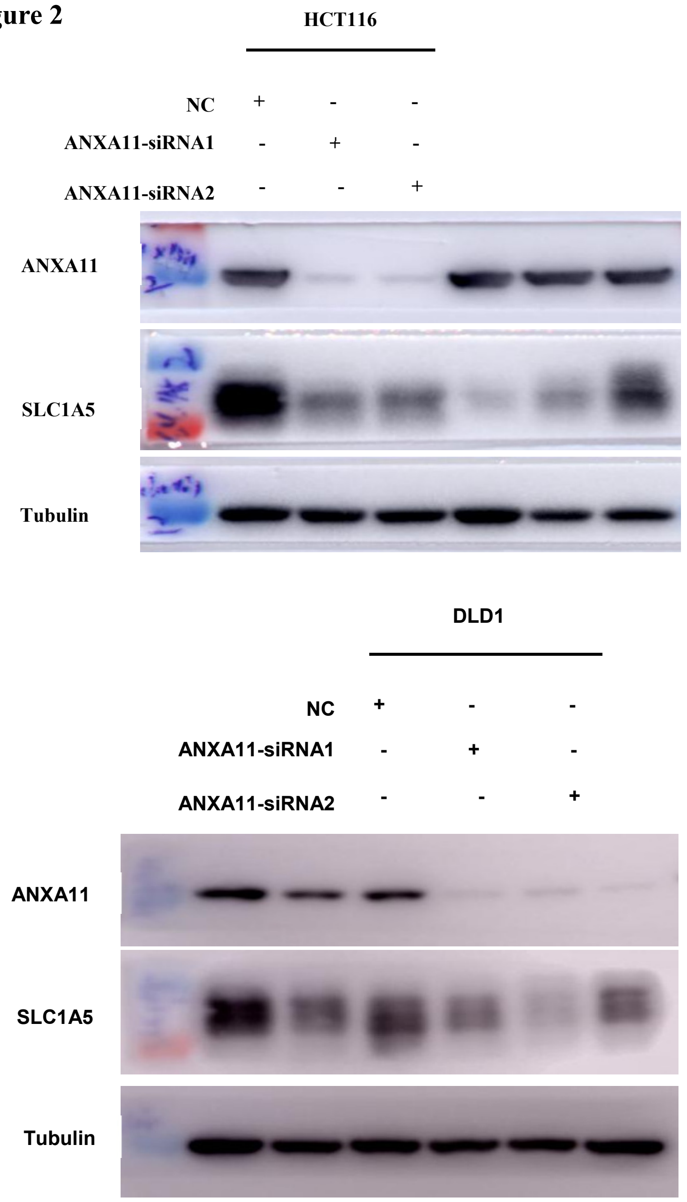
HCT116
NC + - -
ANXA11-siRNA1 - + -
ANXA11-siRNA2 - - +
ANXA11
SLC1A5
Tubulin
DLD1
NC + - -
ANXA11-siRNA1 - + -
ANXA11-siRNA2 - - +
ANXA11
SLC1A5
Tubulin

S-Figure 3

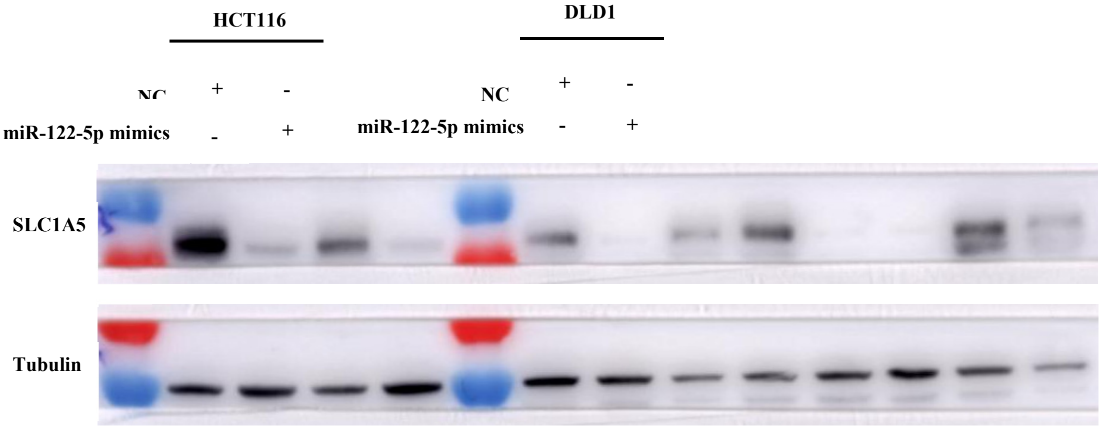

S-Figure 4

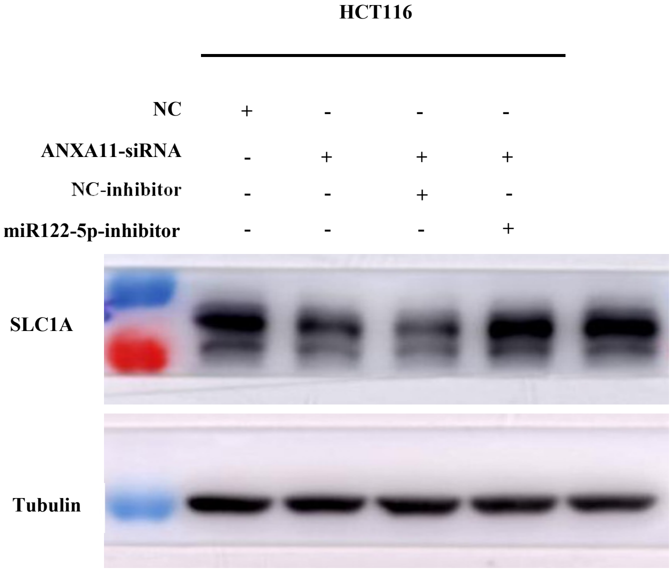

S-Figure 5

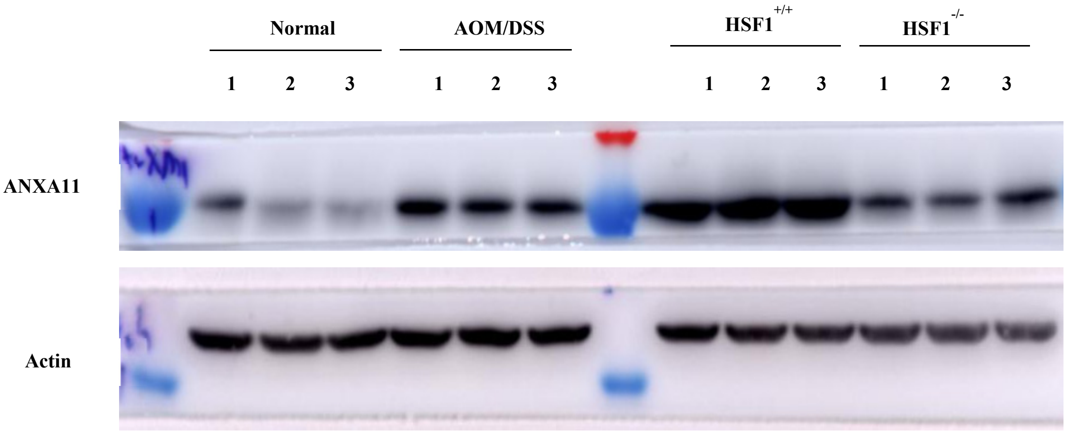

S-Figure 6

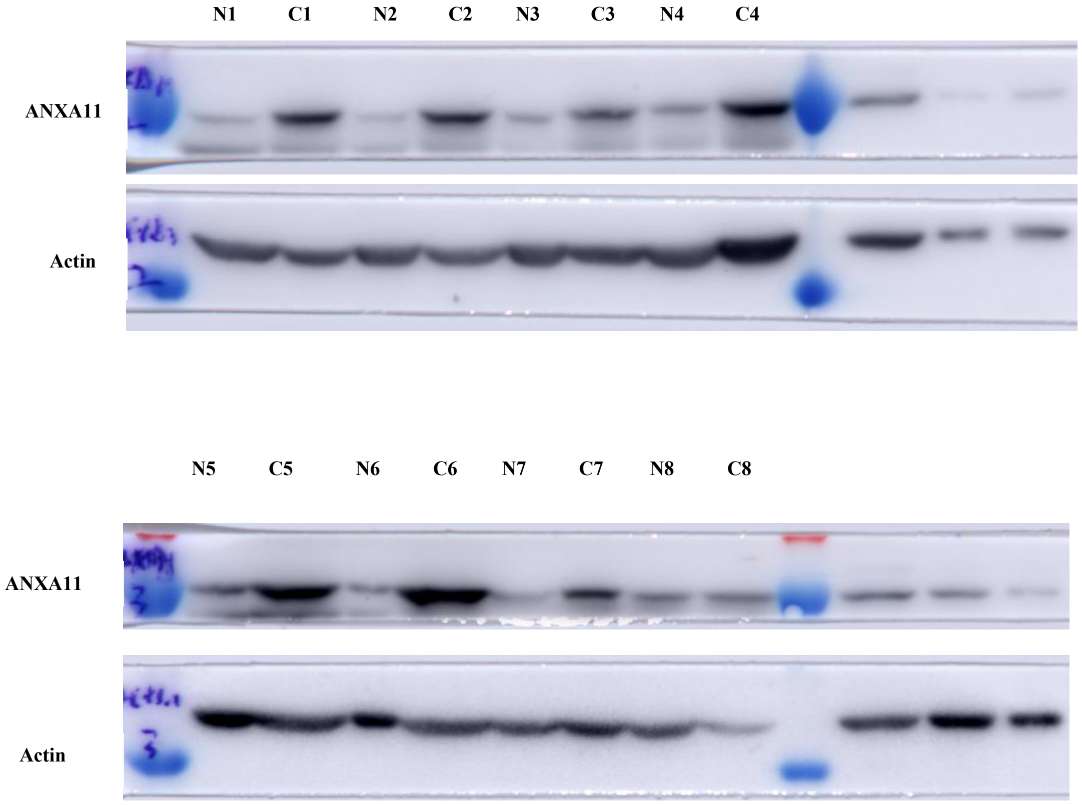

S-Figure 7

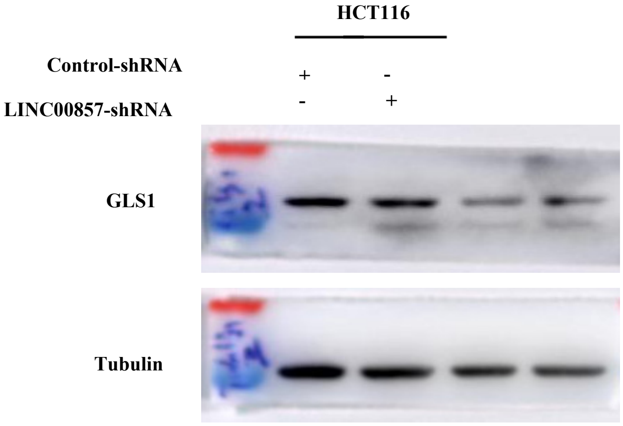

S-Figure 8

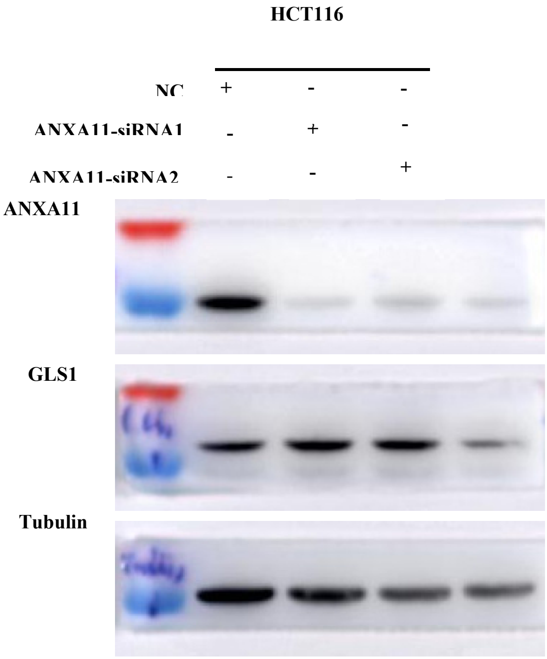

S-Figure 9

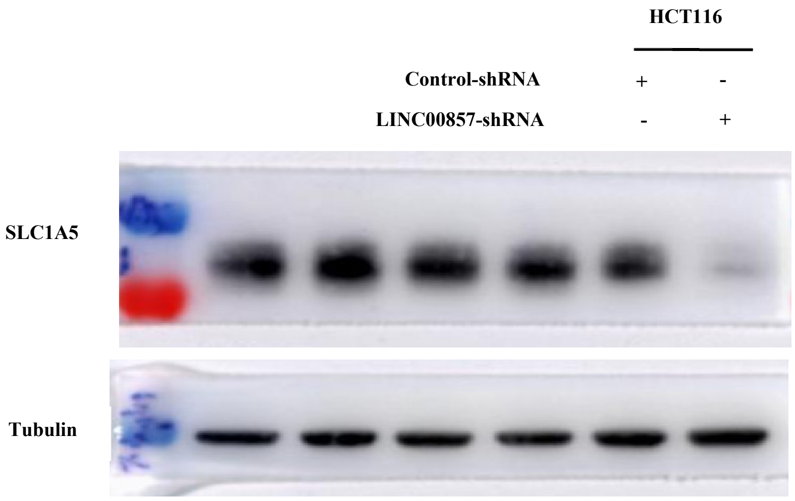
HCT116
Control-shRNA + -
LINC00857-shRNA - +
SLC1A5
Tubulin

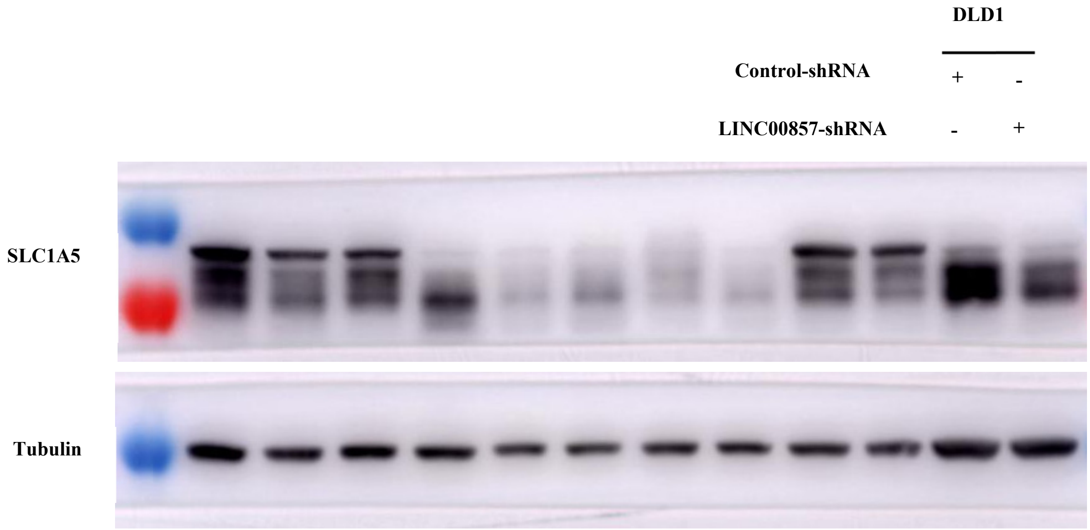
DLD1
Control-shRNA + -
LINC00857-shRNA - +
SLC1A5
Tubulin

S-Figure 10

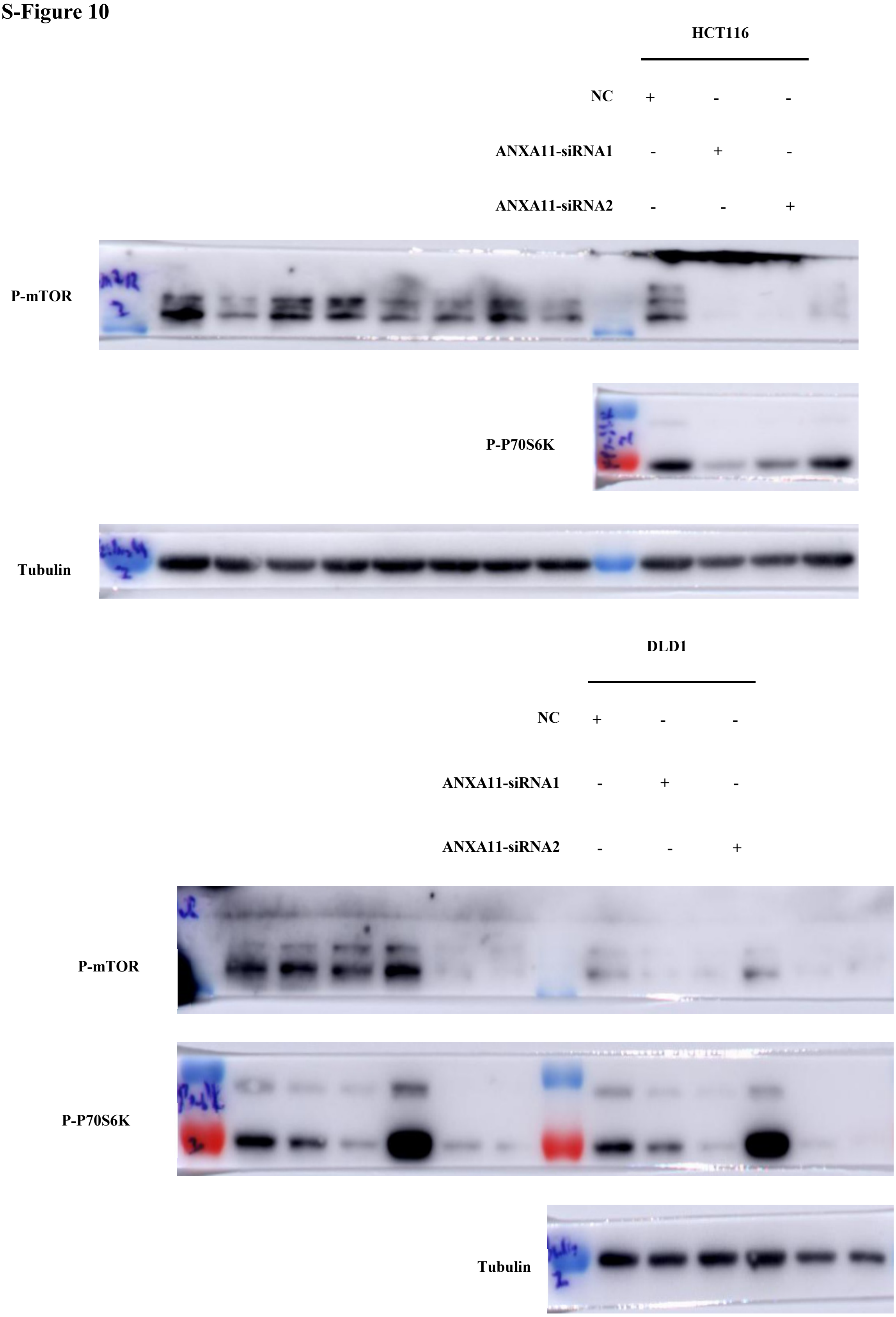
HCT116
NC + - -
ANXA11-siRNA1 - + -
ANXA11-siRNA2 - - +
P-mTOR
P-P70S6K
Tubulin
DLD1
NC + - -
ANXA11-siRNA1 - + -
ANXA11-siRNA2 - - +
P-mTOR
P-P70S6K
Tubulin
